# FOUR-YEAR LONELINESS TRAJECTORY AND ITS PREDICTORS IN OLDER ADULTS

**DOI:** 10.1093/geroni/igae098.2538

**Published:** 2024-12-31

**Authors:** Yan Huang, Lynn Martire, Damon Jones

**Affiliations:** The Pennsylvania State University, University Park, Pennsylvania, United States; The Pennsylvania State University, University Park, Pennsylvania, United States; The Pennsylvania State University, University Park, Pennsylvania, United States

## Abstract

Loneliness is a modifiable risk factor for health decline in older adults and its level may change over time. This study aimed to explore how loneliness changes on average across 4 years in older adults and investigate potential predictors accounting for the formation of this trajectory. Data were collected annually between 2017 to 2020 from 128 older adults (Mage = 77.91) in independent-living or retirement communities in the U.S. Growth curve modeling showed a quadratic model best fit the loneliness trajectory with significant fixed effects of the intercept (b = 2.497, p < 0.001); the linear slope of time (b = -0.036, p = 0.009); and the quadratic term of time (b = 0.002, p < 0.001) as well as three significant random effects (ps < 0.05). The quadratic trajectory showed the level of loneliness decreased first and then increased with individual differences in the intercept and rate of change. We further found being married, having more family and friends, and receiving greater social support at baseline were negatively linked to the overall levels of loneliness. By contrast, living alone, having social strain, depressive symptoms, and difficulty with social participation were positively linked. When including all significant individual variables, only depressive symptoms were still significant (b = 1.850, p < 0.001). Findings suggest the loneliness trajectory follows a nonlinear trend. Baseline relationship and health factors, especially depressive symptoms, are linked to the average level of loneliness at baseline. The COVID-19 pandemic may have influenced the uptick in loneliness in 2020.

